# Supplementary material for: Spectral index-flux relation for investigating the origins of steep decay in γ-ray bursts
Source: Nat Commun. 2021 Jul 7;12:4040. doi: 10.1038/s41467-021-24246-x (PMC8263623; doi:10.1038/s41467-021-24246-x)
Supplement: Supplementary file 1 — Supplementary Information [file 41467_2021_24246_MOESM1_ESM.pdf]

## Supplementary Note 1. HLE from finite-duration pulse.

If we relax the assumption of infinitesimal duration of the pulse (in the jet comoving frame), we can assume that the jet continuously emits until it switches off at a radius $R_0$. For the computation of the flux as a function of time we therefore integrate the comoving intensity along the equal-arrival-time surfaces (EATS)[1,2,3,4]. Photons emitted at different times along the EATS arrive simultaneously to the observer. Knowing that $t_{\text{obs}}(\vartheta) = t_{\text{em}}(1 - \beta \cos\vartheta)$ and imposing that $t_{\text{obs}}(\vartheta, R) = \text{const}$, the polar equation which describes the EATS is given by:

$$R(\vartheta, t_{\text{obs}}) = \frac{\beta c t_{\text{obs}}}{1 - \beta \cos\vartheta} \tag{1}$$

where we have expressed the emission time as $t_{\text{em}} = R/\beta c$, in the assumption of constant expansion velocity. From the above equation, we see that our assumption that the emission switches off when the radius crosses $R_0$ translates to a $\vartheta$-dependent switching off in the observer frame. At any time $t_{\text{obs}} > (1 - \beta)R_0/\beta c$, the observer receives the photons emitted along a surface given by the intersection of the EATS and the jet cone, defined by $R < R_0$, $\vartheta < \vartheta_j$ and $0 < \phi < 2\pi$, where $\phi$ is the azimuth angle. The resulting surface extends from a minimum angle $\vartheta_{\text{min}}(t_{\text{obs}})$ out to $\vartheta = \vartheta_j$, where the former is given by

$$\vartheta_{\text{min}}(t_{\text{obs}}) = \arccos\left(\frac{1}{\beta} - \frac{c t_{\text{obs}}}{R_0}\right) \tag{2}$$

The flux density is given by

$$F_\nu(t_{\text{obs}}) = \int_{\text{EATS}} I_\nu(\vartheta_{\text{obs}}) \cos(\vartheta_{\text{obs}})\, d\Omega_{\text{obs}} \tag{3}$$

where $I_\nu$ is the specific intensity and $d\Omega_{\text{obs}}$ is the solid angle in the observer frame. Transforming to the comoving frame we have $I_\nu(\nu) = \mathcal{D}^3 I'_{\nu'}(\nu/\mathcal{D})$. We decompose the comoving intensity as

$$I'_{\nu'} = I'_{\nu'_p} \cdot S_{\nu'} \tag{4}$$

where $I'_{\nu'_p}$ is the comoving intensity at the peak frequency $\nu'_p$ and $S_{\nu'}$ is the comoving spectral shape, normalized so that $S_{\nu'}(\nu'_p) = 1$. In general, $I'_{\nu'_p} \propto N_{\text{tot}}/R^2$, where $N_{\text{tot}}$ is the number of emitting particles. If the emission process is synchrotron, $I'_{\nu'_p}$ is also proportional to $B$, the magnetic field as measured by an observer comoving with the jet, which is assumed to evolve as $B = B_0(R/R_0)^{-\lambda}$, with $\lambda \geq 0$ is a free parameter. If we assume $N_{\text{tot}}$ to be constant in time, then $I'_{\nu'_p} \propto R^{-2}$ and, since $d\Omega_{\text{obs}} \propto R^2 \sin\vartheta$, the final form of the integral is

$$F_\nu(t_{\text{obs}}) \propto \int_{\vartheta_{\text{min}}(t_{\text{obs}})}^{\vartheta_j} S_{\nu'}(\nu/\mathcal{D}(\vartheta)) \left(\frac{R(\vartheta, t_{\text{obs}})}{R_0}\right)^{-\lambda} \mathcal{D}^3(\vartheta) \sin\vartheta \cos\vartheta d\vartheta \tag{5}$$

The $\alpha - F$ relation for several values of $\lambda$ is plotted in Supplementary Fig. 1.

**Supplementary Note 2. HLE from an accelerating shell.**

In this section we test the effect of relaxing the assumption that the shell which generates HLE expands with a constant bulk Lorentz factor $\Gamma$[5,6,7,8]. For our treatment we consider that the emission starts at $R = R_{\rm in}$ and finishes at $R = R_{\rm off}$. We assume also that $\Gamma$ evolves as a power law with the radius, namely

$$\Gamma(R) = \Gamma_0 \left(\frac{R}{R_{\rm in}}\right)^k \tag{6}$$

with $k > 0$ if the shell accelerates or $k < 0$ if the shell decelerates. We consider the emission of a photon at radius $R_{\rm em}$ and an angle $\vartheta = \vartheta_{\rm em}$, then we define $\Delta t_{\rm em}$ the time necessary to expand from $R_{\rm in}$ to $R_{\rm em}$. During the same interval of time, a photon emitted at radius $R_{\rm in}$ and an angle $\vartheta = 0$ travels a distance $c\Delta t_{\rm em}$. Therefore the delay between these two photons is $\Delta t_{\rm obs} = (R_{\rm in} + c\Delta t_{\rm em} - R_{\rm em}\cos\vartheta)/c$. From eq. (6) we can write

$$\frac{1}{\sqrt{1 - \frac{1}{c^2}\left(\frac{dR}{dt}\right)^2}} = \Gamma_0 \left(\frac{R}{R_{\rm in}}\right)^k \tag{7}$$

from which we derive

$$\frac{dR}{\sqrt{1 - \frac{1}{\Gamma_0^2}\left(\frac{R}{R_{\rm in}}\right)^{-2k}}} = c\,dt \tag{8}$$

In the limit of $\Gamma_0 \gg \left(\frac{R}{R_{\rm in}}\right)^{-k}$, we can write

$$\int_{R_{\rm in}}^{R_{\rm em}} \left[1 + \frac{1}{2\Gamma_0^2}\left(\frac{R}{R_{\rm in}}\right)^{-2k}\right] dR \simeq c\Delta t_{\rm em} \tag{9}$$

Thus, the delay time is

$$\Delta t_{\rm obs} = \frac{R_{\rm em}}{c}(1 - \cos\theta) + \frac{1}{2c}\int_{R_{\rm in}}^{R_{\rm em}} \frac{1}{\Gamma^2} dR \tag{10}$$

Given an arrival time $\Delta t_{\rm obs}$, this equation allow us to associate a radius $R_{\rm em}$ to each angle $\vartheta_{\rm em}$ through the following expression:

$$\cos\vartheta_{\rm em} = 1 - \frac{c\Delta t_{\rm obs}}{R_{\rm em}} + \frac{R_{\rm in}}{2R_{\rm em}}\frac{1}{\Gamma_0^2}\frac{1}{1-2k}\left[\left(\frac{R_{\rm em}}{R_{\rm in}}\right)^{1-2k} - 1\right] \tag{11}$$

Inverting this equation, we obtain the polar equation $R_{\rm em}(\vartheta_{\rm em}, \Delta t_{\rm obs})$ which defines the EATS, namely all the photons emitted from this locus of points arrive to the observer with a time delay $\Delta t_{\rm obs}$ with respect to the first photon coming from $R = R_{\rm in}$ and $\vartheta = 0$. The computation of the flux as a function of time is again done using eq. (5), with the only difference that now $\beta$ and $\Gamma$, which

appear in the Doppler factor $\mathcal{D}(\vartheta)$, depend on $R(\vartheta)$. The light curve and the spectral evolution for values of $k$ in the range $-0.4 \leq k \leq 0.4$ are shown in Supplementary Fig. 2.

**Supplementary Note 3. Alternative scenarios shaping the X-ray tails.**

In this section we explore other possible models of prompt emission which can drive the evolution during the X-ray tails. We can consider, for instance, an anisotropic emission from the jet core, made of mini-jets[10,11,12] with angular sizes $< 1/\Gamma$. In order to model such anisotropy we adopt an angular distribution of the emission in the form $P(\theta') \propto (\sin\theta')^n$, where $n$ is the degree of anisotropy and $\theta'$ is the angle between the direction of the emitted photons and the local radial direction, as measured in the comoving frame. We evaluate the resulting HLE flux received by the observer as

$$F_\nu \propto P(\theta')\mathcal{D}^2(\theta) S\left(\frac{\nu}{P(\theta')\mathcal{D}(\theta)\nu'_c}\right) \tag{12}$$

where the dependence on time is implicit in $\theta$. The resulting $\alpha - F$ relation for $n = 2$, $n = 5$ and $n = 10$ is shown in Supplementary Fig. 4. The figure demonstrates that the larger the value of $n$, the more the predicted curves move away from the data. Since for $n = 0$ we are in the limit of standard HLE, which is already disfavoured by our study, we conclude that also mini-jets are not able to successfully reproduce the $\alpha$ - F relation.
Within the HLE scenario, only models which assume a dissipation occurring above the jet photosphere, such as in internal shocks[13] or in magnetic reconnection scenarios[9,14], are able to reproduce the typical duration of X-ray tails ($\sim$ 100 s). Photospheric models[15], where dissipation occurs at radii $R_{\rm ph} \sim 10^{12}$ cm[16], give smaller times scales of $\sim 10^{-2}$ s, incompatible with observations. Only a common declining activity of the central engine[17,18] and a fine-tuned intrinsic spectral softening[19] would be required to account for the $\alpha - F$ relation.
In slow heating/reacceleration scenarios[20], as soon as the shock crosses the shell, particle acceleration is halted along with the generation of magnetic field (as both rely on the presence of shock-generated turbulence), leading to an abrupt switch-off of the emission. This leads again to HLE being the dominant effect in determining the tail flux and spectral evolution, which is clearly disfavoured by our analysis. A slower decay of the magnetic field after the shock crossing[21], along with a decaying particle acceleration, could be compatible with our results, but we still need adiabatic cooling (which is anyway unavoidable) to play the leading role in the spectral evolution, as discussed in the next section. We therefore conclude that, while the slow-heating scenario is not *per se* rejected by our results, it cannot be invoked as the main mechanism behind the $\alpha$ - F relation.

**Supplementary Note 4. Possible temporal evolution of the comoving spectral shape.**

In the derivation of spectral evolution from adiabatic cooling, we implicitly assumed that we are in the early post-prompt phase, namely where no more particles are injected/accelerated and the particle distribution only evolves according to the cooling processes. If adiabatic cooling is

dominant, the energy of all particles evolve at the same way, according to the following equation:

$$\gamma^3 V' = \text{const} \tag{13}$$

where $\gamma$ is the Lorentz factor of the particle and $V'$ is the comoving volume. Therefore the shape of the particle distribution, and hence of the spectrum, does not change in time, but is only rigidly shifted at lower energies.

If adiabatic and radiative cooling are competing on comparable timescales, in the post-prompt phase an exponential cutoff appears above the cooling energy $\gamma_c$. If also the magnetic field decays, the adiabatic cooling tends to dominate with time and also the cutoff energy would eventually evolve according to eq. 13, going again in the limit of rigidly shifted spectrum.

The assumption of rigidly shifted spectrum might not hold if the particle injection gradually decreases in time, instead of ceasing abruptly. The temporal evolution of particle distribution in case of decreasing injection of particles is studied solving numerically the cooling equation

$$\frac{\partial N}{\partial t} = \dot{N}_{\text{inj}} - \frac{\partial}{\partial \gamma}(N\dot{\gamma}) \tag{14}$$

where $N(\gamma) = dN_{\text{e}}/d\gamma$ ($N_{\text{e}}$ being the number of emitting particles). The resulting evolution of the synchrotron spectrum is shown in Supplementary Fig. 5a, where we assumed an injection term of the form $\dot{N}_{\text{inj}} \propto (t/t_{\text{inj}})^{-y}$, with $y > 0$, and a constant magnetic field. Imposing that, at the beginning, the $F_\nu$ peak is just above the observing band, the resulting spectral evolution would be an initial softening followed by a hardening, as shown in Supplementary Fig. 5b. In the case of a decay of both the magnetic field and $\dot{N}_{\text{inj}}$, the corresponding effects on the spectral shape tend to compensate each other[22], giving a bare modification of the spectral shape or even a hardening (see Supplementary Fig. 6). In conclusion, intrinsic modifications of the spectral shape can hardly give an agreement with data comparable to the case of a rigidly shifting spectrum.

**Supplementary Note 5. Supplementary discussion.**

As stated in the main text, HLE and adiabatic cooling have the same timescale, but the relevance of one process with respect to the other is determined by the decay of the magnetic field, which governs the drop of the spectrum normalization. The expected value of $\lambda$ can be derived in several scenarios, according to the process that rules the magnetic field evolution. In case of conservation of magnetic flux, the perpendicular and parallel component of $B$ evolve as $B_\perp \sim 1/(\Delta R' \cdot r)$ and $B_{//} \sim r^{-2}$, where $r$ is the transverse radial dimension of the jet in a cylindrical reference system $(r, \phi, z)$. If the jet is conical then $r \propto R$, leading to $B \sim R^{-1}$ ($\lambda = 1$) for $\Delta R' = \text{const}$ and $B \sim R^{-2}$ ($\lambda = 2$) for $\Delta R' \propto R$. Another possibility predicts equipartition between magnetic energy density and particle energy density, giving $B^2 \sim \langle\gamma\rangle/V \sim V^{-4/3}$, where in the last step we used eq. (13). In this case $B \sim R^{-4/3}$ ($\lambda = 4/3$) for $\Delta R' = \text{const}$ and $B \sim R^{-2}$ ($\lambda = 2$) for $\Delta R' \propto R$. All these predicted values of $\lambda$ are larger than the range found from our analysis. Such tension can be solved, for instance, if the shell thickness decreases as the jet expands, or if the jet is not conical (e.g. paraboloidal, with $r \propto \sqrt{R}$).

## Supplementary Figures

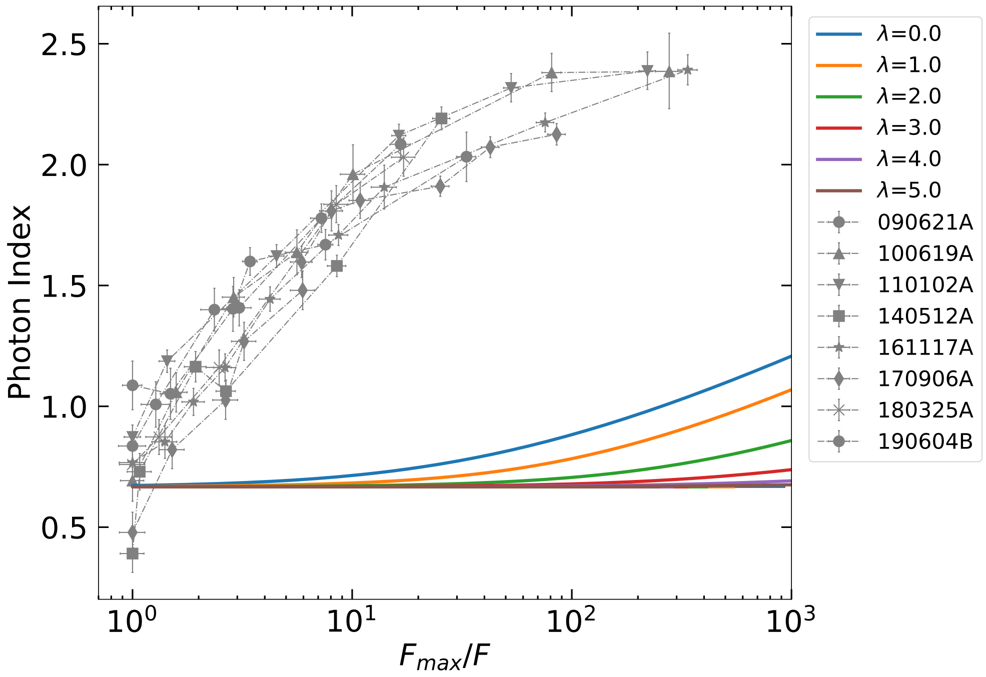


**Supplementary Fig. 1: Spectral evolution in case of HLE from a finite-duration pulse.** The adopted parameters are $R_{\rm in} = 3 \times 10^{15}$ cm, $R_{\rm off} = 9 \times 10^{15}$ cm, $\Gamma_0 = 100$ and $\nu_p = 100$ keV. The adopted spectral shape is a SBPL. The value of $\lambda$ specifies the evolution of the magnetic field. The error bars represent $1\sigma$ uncertainties, calculated via spectral fitting in XSPEC. In the legend we report the name of each GRB.

**a**

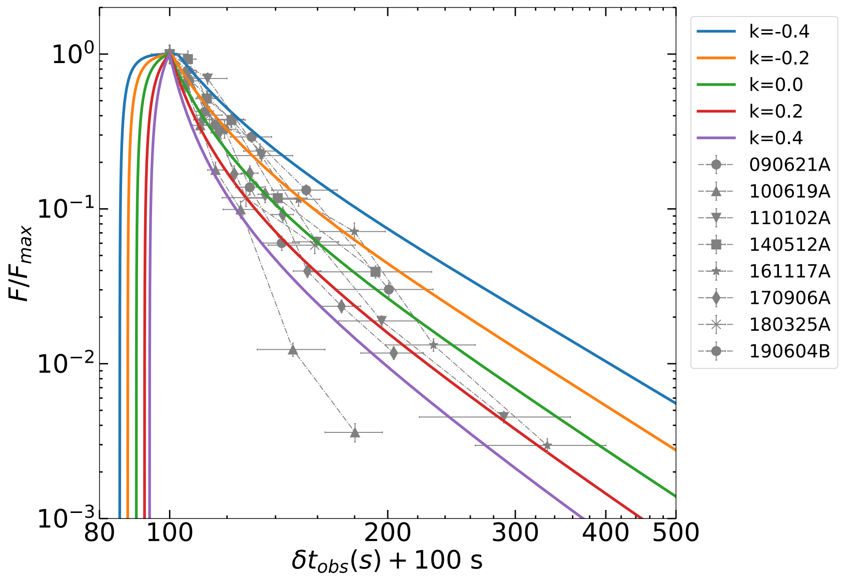


**b**

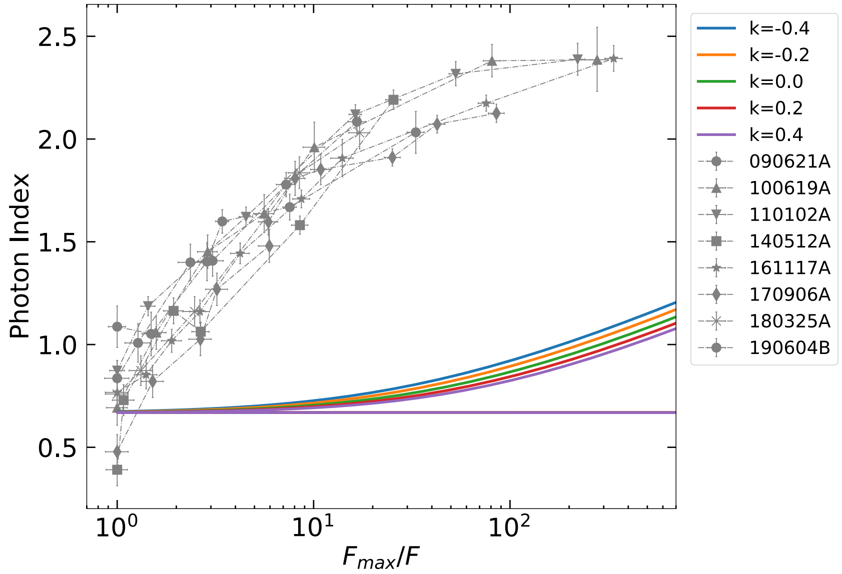


**Supplementary Fig. 2: Temporal (a) and spectral (b) evolution for HLE from a finite-duration pulse, in case of not constant $\Gamma$.** The magnetic field does not evolve with radius, i.e $\lambda = 0$. The adopted parameters are $R_{\rm in} = 3 \times 10^{15}$ cm, $R_{\rm off} = 9 \times 10^{15}$ cm, $\Gamma_0 = 100$ and $\nu_p = 100$ keV. The value of $k$ specifies the evolution of $\Gamma$. The adopted spectral shape is a SBPL. The peak of each curve is shifted at 100 s. In **a** the vertical error bars represent $1\sigma$ uncertainties, calculated via spectral fitting in XSPEC, while horizontal error bars represent the width of the time bin. In **b** the error bars represent $1\sigma$ uncertainties, calculated via spectral fitting in XSPEC. In the legend we report the name of each GRB.

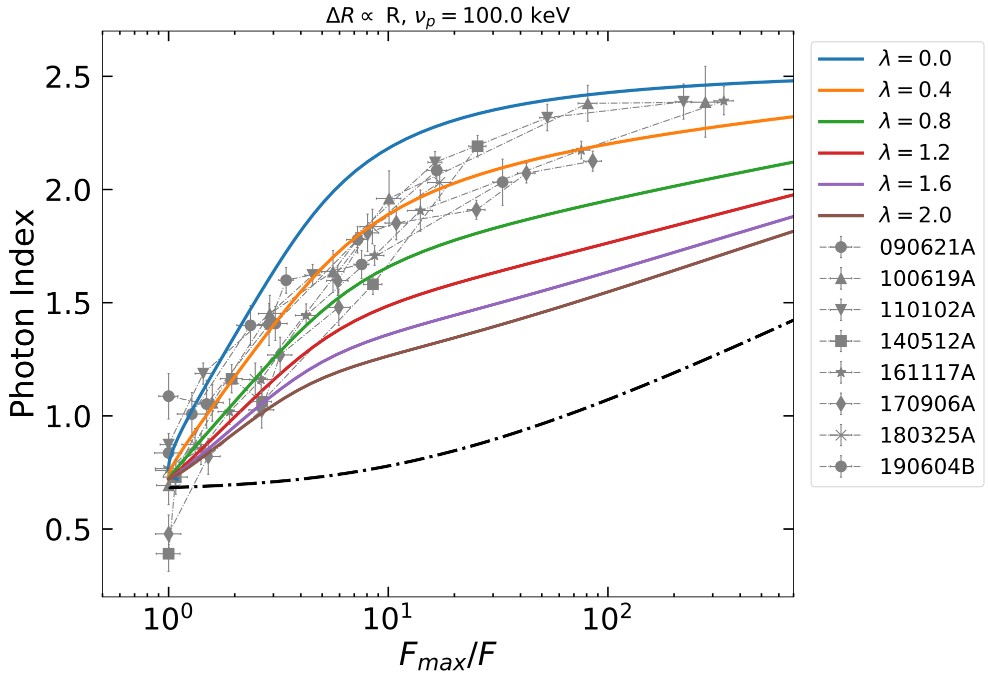


**Supplementary Fig. 3:** $\alpha - F$ **plot in case of adiabatic cooling, but with the shell thickness** $\Delta R \propto R$**, instead of** $\Delta R =$ **const.** The theoretical curves are computed taking also into account the effect of HLE. The value of $\lambda$ specifies the evolution of the magnetic field. We adopt a SBPL as spectral shape with $\alpha_{\rm s} = -1/3$ and $\beta_{\rm s} = 1.5$, an initial observed peak frequency of 100 keV. The dot-dashed line is the evolution expected in case of HLE without adiabatic cooling, assuming the same spectral shape and initial observed peak frequency. The error bars represent $1\sigma$ uncertainties, calculated via spectral fitting in XSPEC. In the legend we report the name of each GRB.

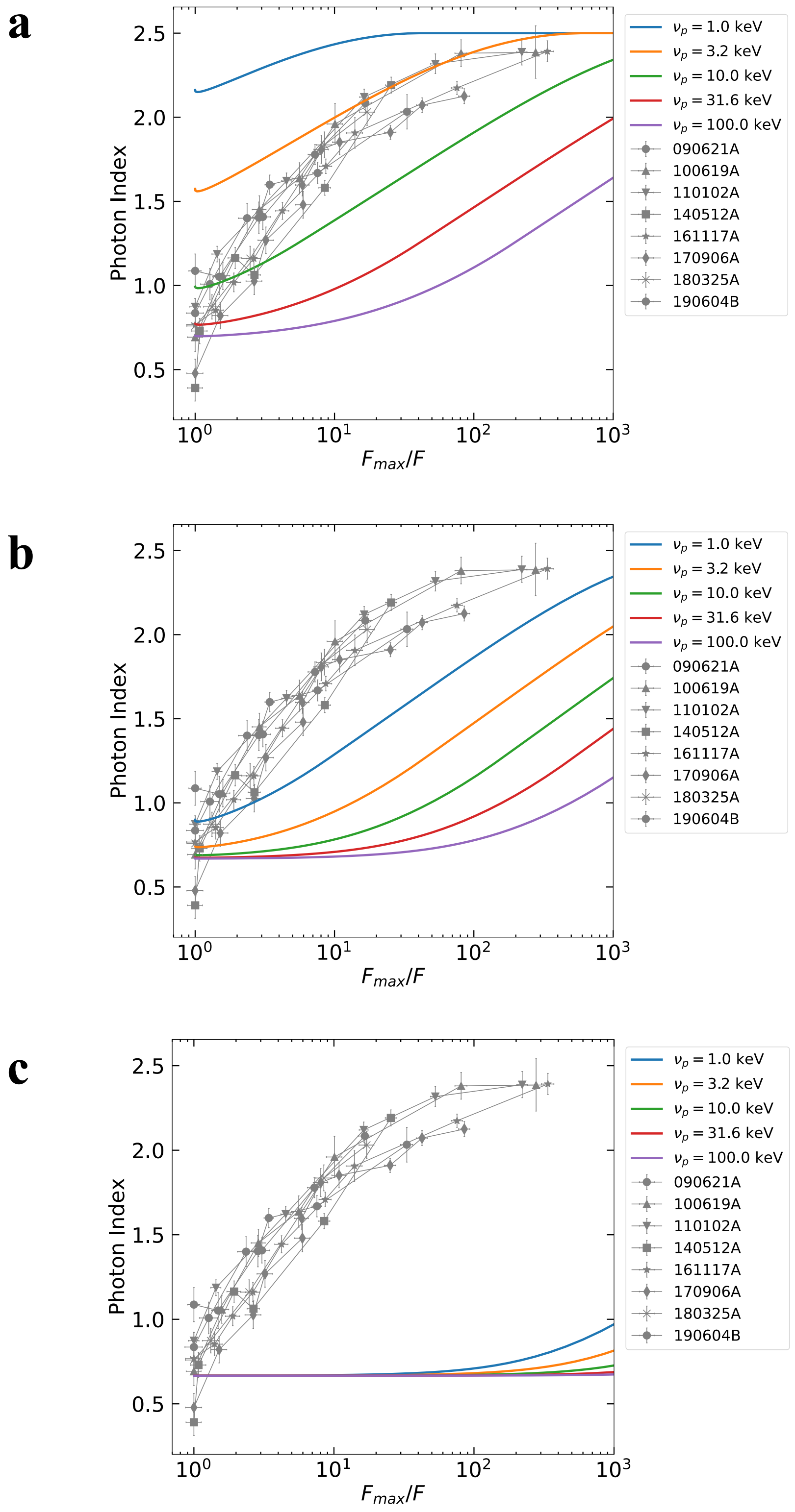


**Supplementary Fig. 4: Predicted $\alpha - F$ relation in case of mini-jets model**. We show the evolution for $n = 2$ (**a**), $n = 5$ (**b**) and $n = 10$ (**c**). The error bars represent $1\sigma$ uncertainties, calculated via spectral fitting in XSPEC. In the legend we report the name of each GRB.

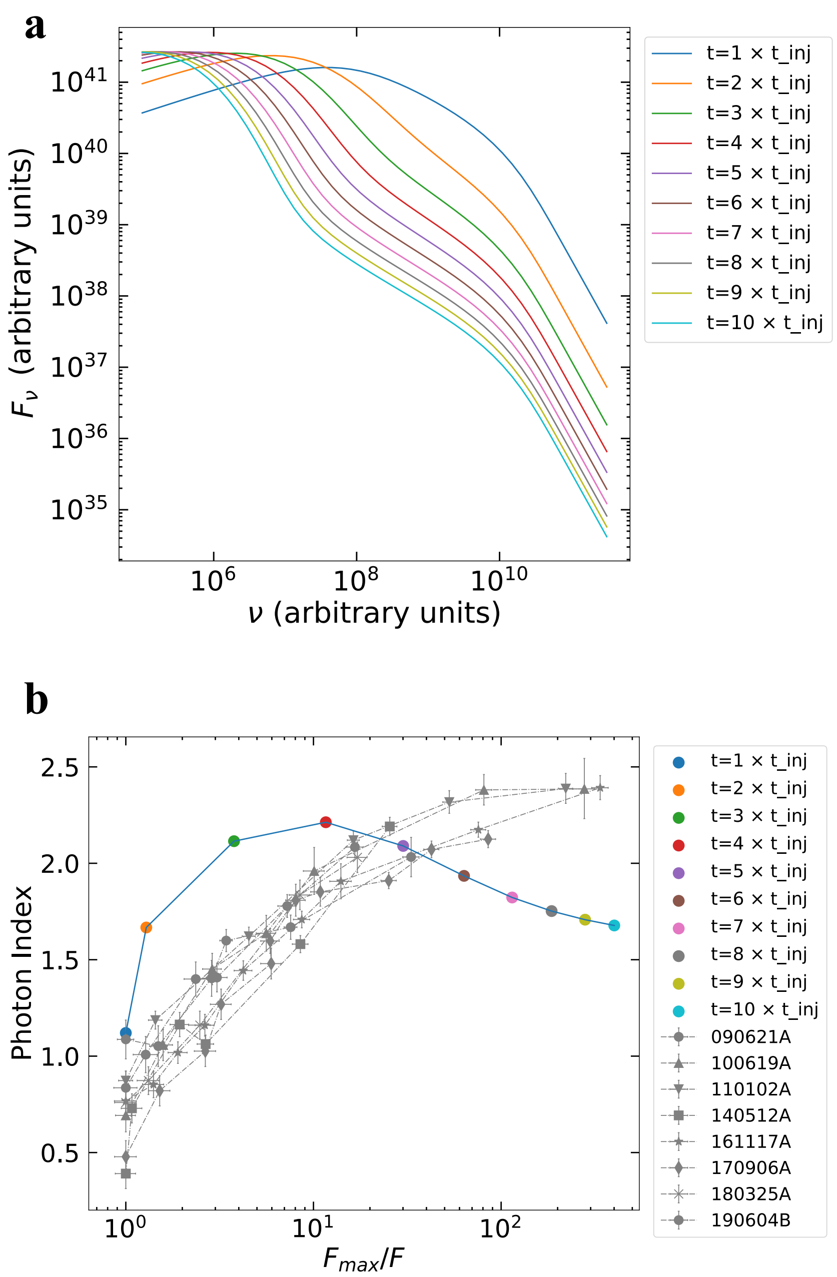


**Supplementary Fig. 5: Spectral evolution of the synchrotron spectral shape for a decaying particle injection**. **a** shows how the spectral shape evolves, adopting a decaying index for the injection rate $y = 3$ and a constant magnetic field. The time goes from $t = t_{\rm inj}$ (blue line) up to $t = 10 \times t_{\rm inj}$ (cyan line), with steps of $t_{\rm inj}$. In panel **b** the blue line shows the corresponding $\alpha - F$ relation imposing that the observing band is below the initial spectral peak, which ensures that the initial photon index is $\sim 2/3$. With different coloured points we indicate the evolution in the $\alpha - F$ plane as a function of time, from $t = t_{\rm inj}$ (blue point) up to $t = 10 \times t_{\rm inj}$ (cyan point), with steps of $t_{\rm inj}$. The error bars represent $1\sigma$ uncertainties, calculated via spectral fitting in XSPEC.

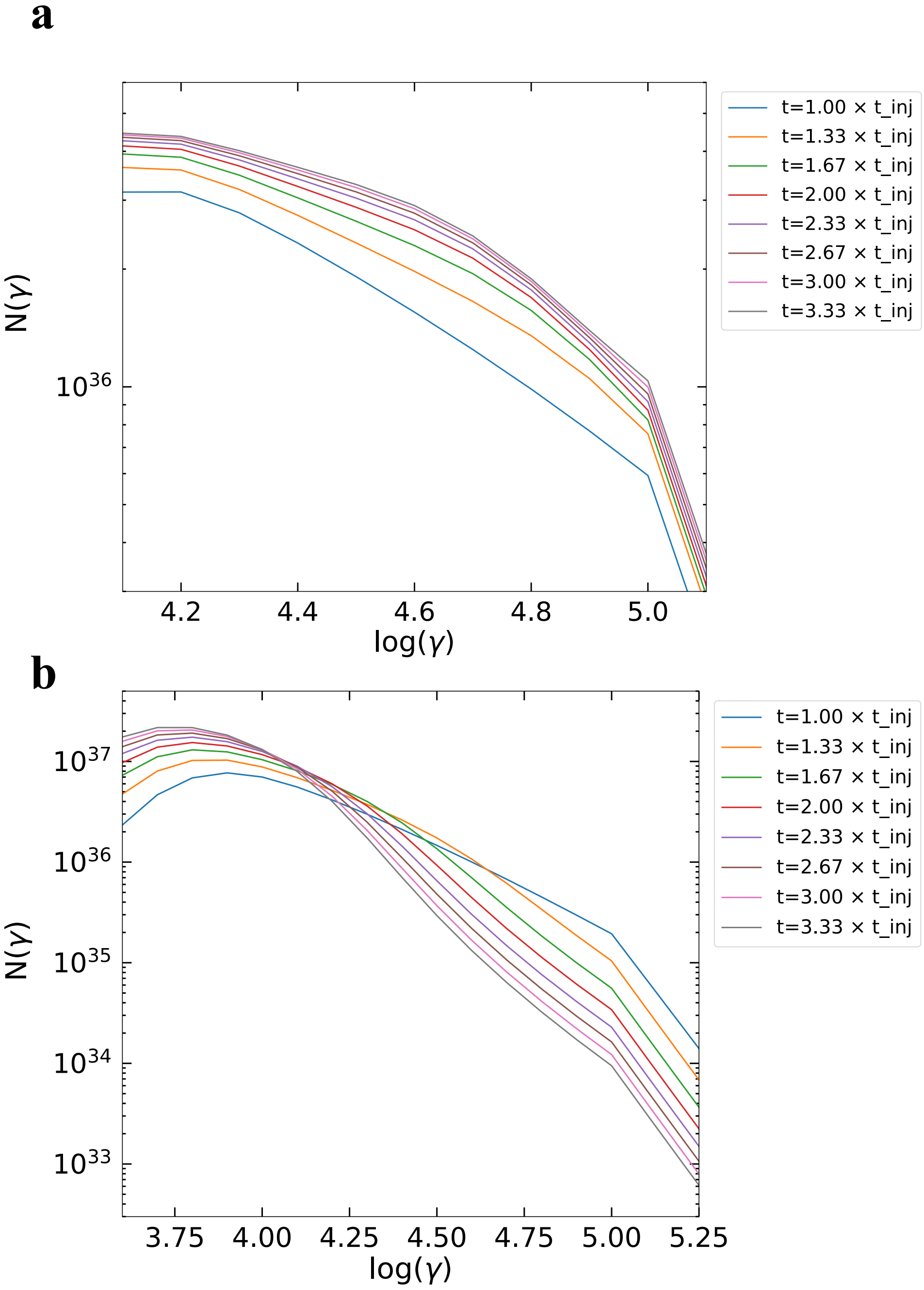


**Supplementary Fig. 6: Temporal evolution of the particle distribution for a decay of both $\dot{N}_{\mathbf{inj}}$ and magnetic field**. The adopted parameters are $y = 2$ and $\lambda = 2$ for **a**, $y = 4$ and $\lambda = 1$ for **b**. The evolution is followed from $t = t_{\rm inj}$ (blue line), which is the standard $\gamma^{-2}$ cooling branch of the distribution) up to $t = 3.33 \times t_{\rm inj}$ (grey line), with steps of $\sim 4/3\, t_{\rm inj}$.

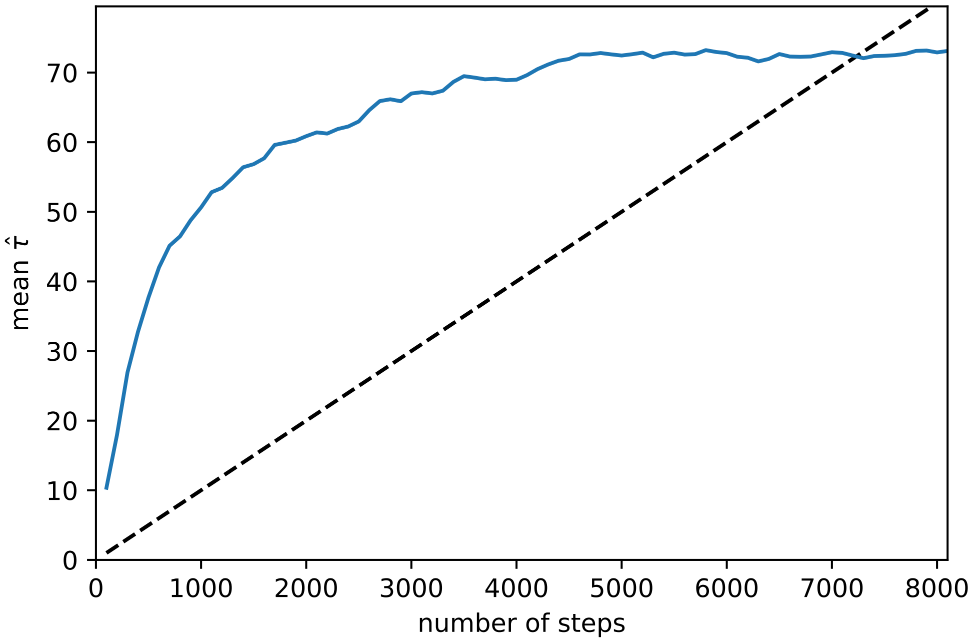


**Supplementary Fig. 7: An example of autocorrelation time-MCMC steps plot (GRB 161117A)**. The blue line indicates how $\hat{\tau}$, the autocorrelation time (adimensional quantity), evolves as a function of the number of MCMC steps. The dashed line corresponds to number of steps $= 100 \times \hat{\tau}$.

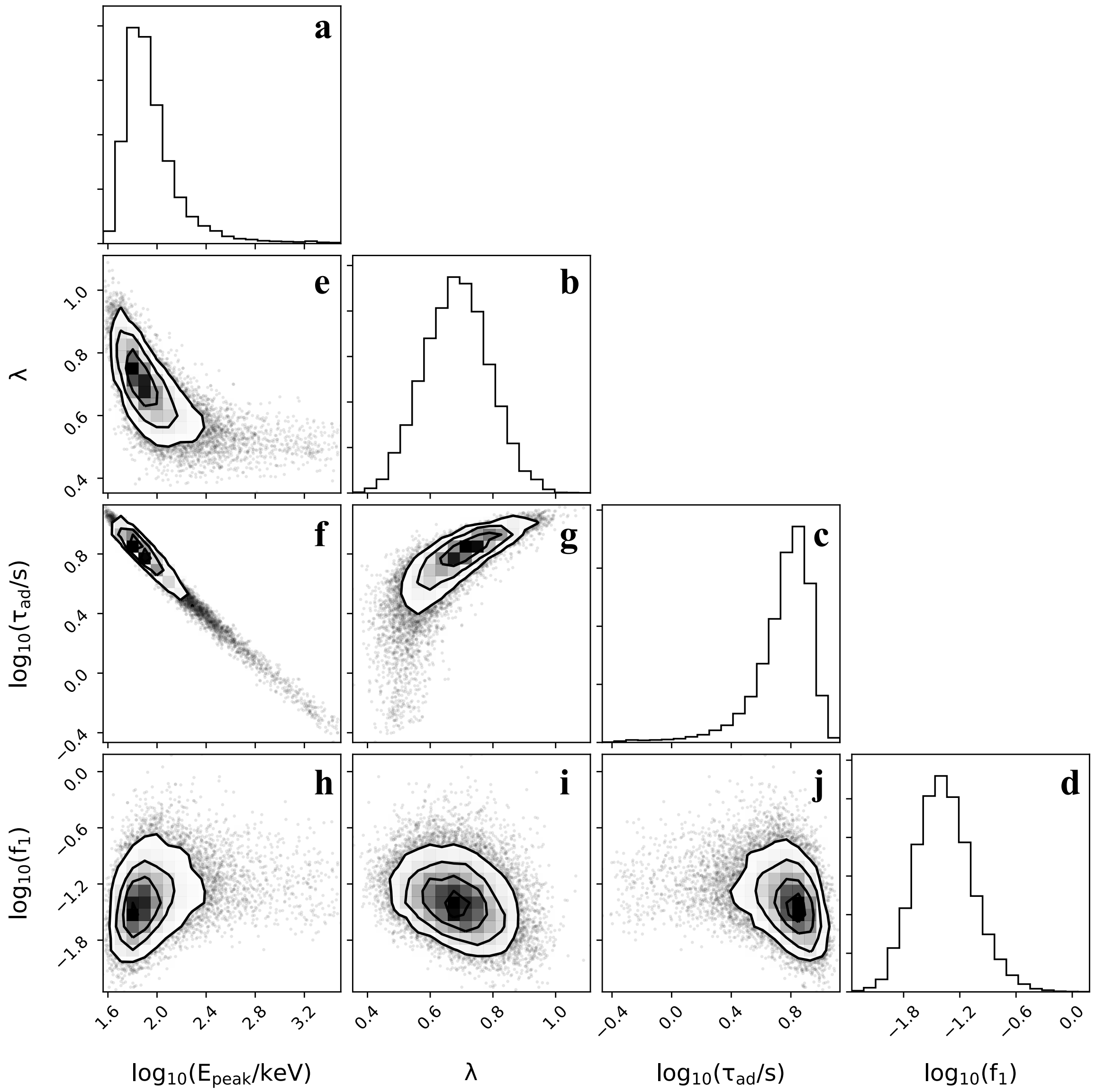


**Supplementary Fig. 8: An example of corner plot from the MCMC (GRB 161117A).** $E_{\rm peak}$ is the peak energy at the beginning of the steep decay, $\lambda$ is the decaying index of magnetic field (adimensional parameter), $\tau_{\rm ad}$ is the adiabatic timescale, $f_1$ is a parameter used in the definition of the likelihood (see the methods section in the main text for further details). The panels **a-d** show the 1D posterior probability distribution of each parameter; since the y axis is a measure of probability density, it has an arbitrary scale. The panels **e-j** show the 2D posterior distribution for each couple of parameters and the contour lines represent the confidence regions at $0.5\sigma$, $1\sigma$, $1.5\sigma$ and $2\sigma$ level of confidence (if only 3 contours are visible, this means that the inner one, corresponding to $0.5\sigma$, is so small that it is reduced to a point and is not shown).

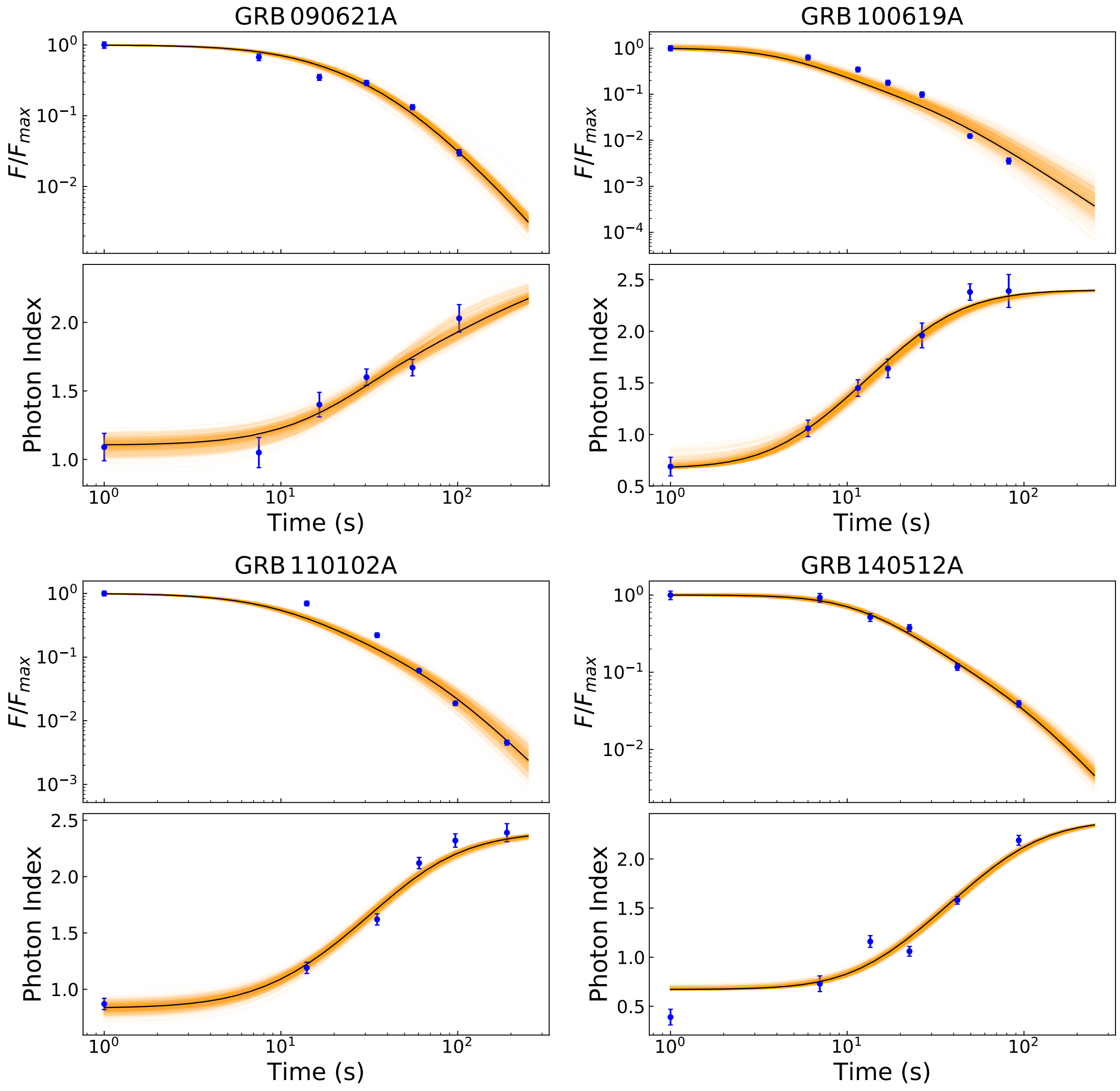


**Supplementary Fig. 9: Joint temporal evolution of normalized flux and photon index.** For each GRB we compare the data (blue points) with the best fit curve of the adiabatic cooling model (black line). The orange lines are curves produced extracting randomly the model parameters from the posterior distribution obtained from the MCMC. 500 lines are plotted together and their superposition creates a confidence band of the model. In some regions of the plot the band appears narrower because the parameters uncertainty produces a smaller scatter of the lines. The error bars represent $1\sigma$ uncertainties and they are derived from spectral analysis.

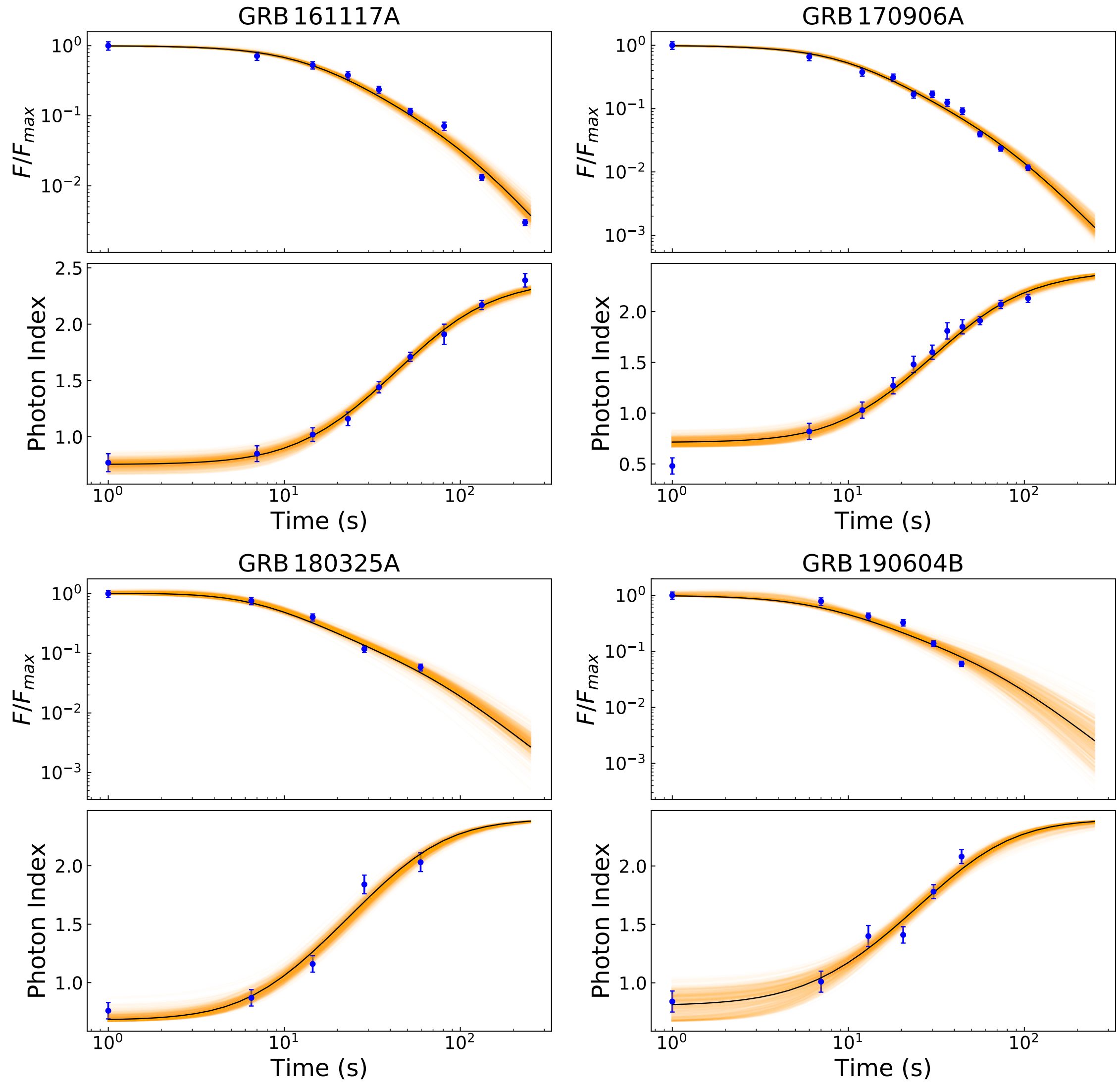


**Supplementary Fig. 10: Joint temporal evolution of normalized flux and photon index-continued.** For each GRB we compare the data (blue points) with the best fit curve of the adiabatic cooling model (black line). The orange lines are curves produced extracting randomly the model parameters from the posterior distribution obtained from the MCMC. 500 lines are plotted together and their superposition creates a confidence band of the model. In some regions of the plot the band appears narrower because the parameters uncertainty produces a smaller scatter of the lines. The error bars represent $1\sigma$ uncertainties and they are derived from spectral analysis.

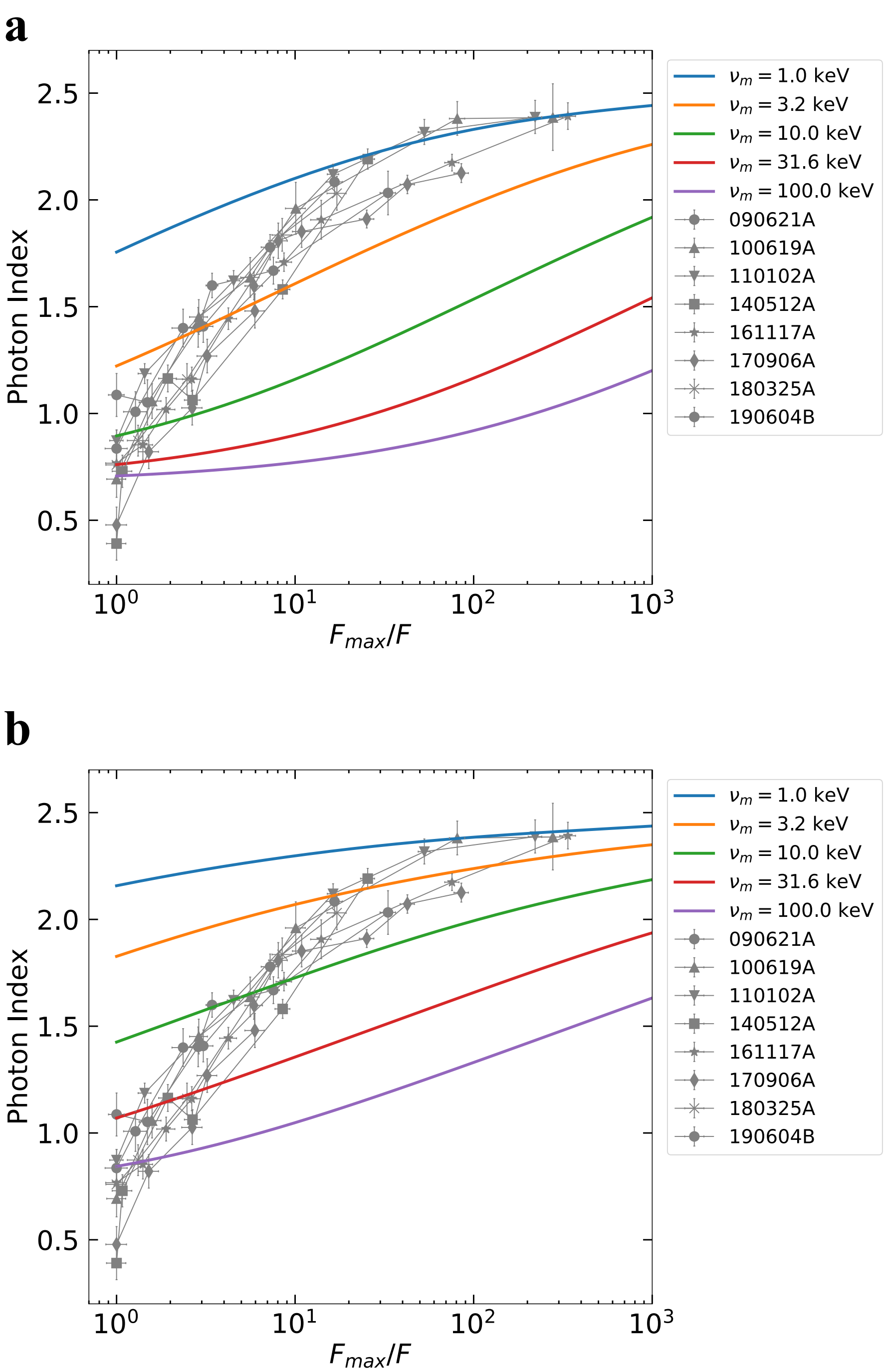


**Supplementary Fig. 11: Spectral evolution expected for HLE from a infinitesimal duration pulse, assuming a synchrotron spectrum as spectral shape.** In **a** we adopt $\nu_{\rm m}/\nu_{\rm c} = 1$, while in **b** $\nu_{\rm m}/\nu_{\rm c} = 10$. The several colors indicate the observed peak frequency at the beginning of the decay. In **b** the spectral evolution appears slightly steeper with respect to the case $\nu_m/\nu_c = 1$ because for $\nu_{\rm c} < \nu < \nu_{\rm m}$ the spectrum goes like $F_\nu \sim \nu^{-p/2}$. The error bars represent $1\sigma$ uncertainties, calculated via spectral fitting in XSPEC. In the legend we report the name of each GRB.

**a**

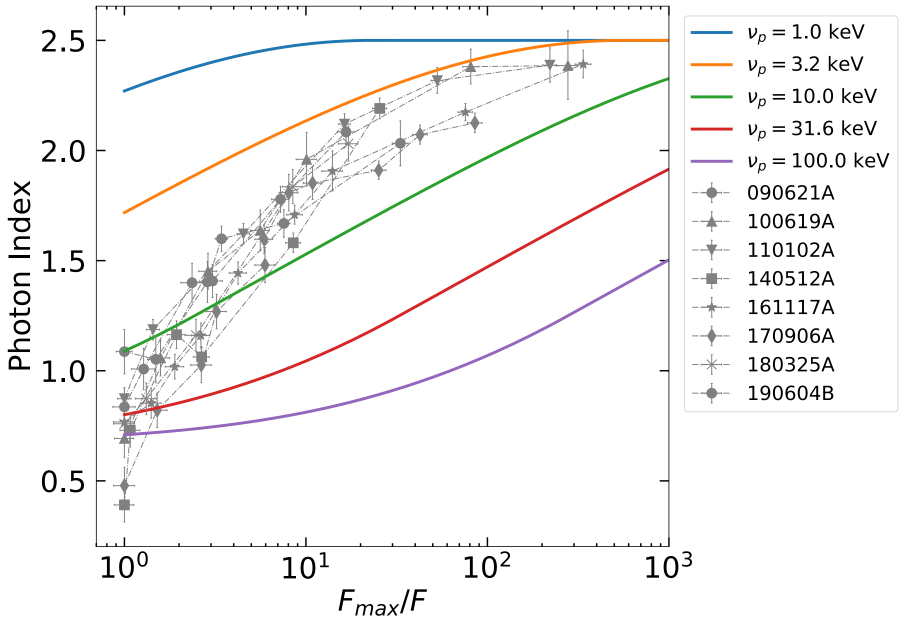


**b**

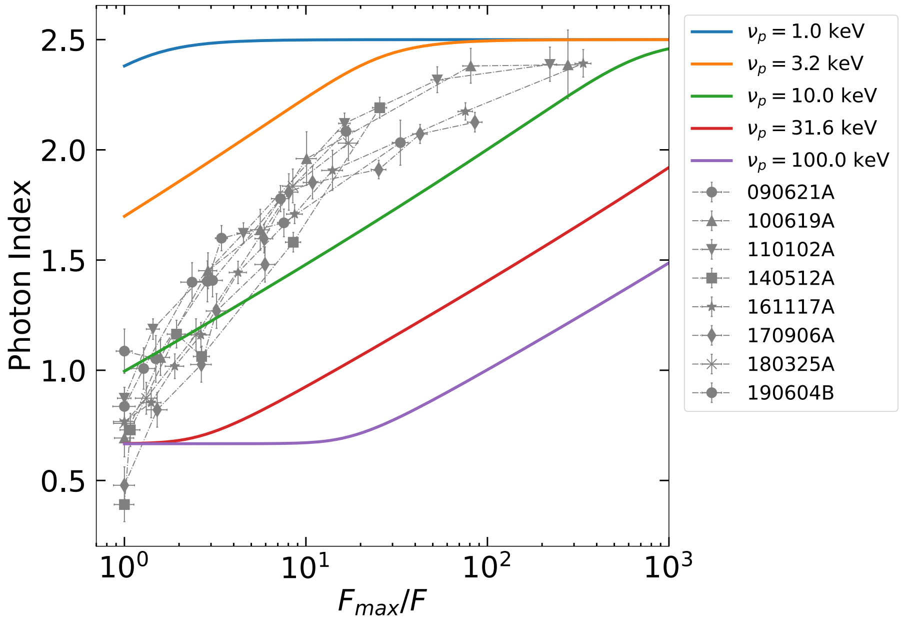


**Supplementary Fig. 12: Spectral evolution expected for HLE from a infinitesimal duration pulse, for alternative spectral shapes.** In **a** we adopt a Band function, while in **b** we adopt a SBPL with sharpness parameter $n = 4$. The error bars represent $1\sigma$ uncertainties, calculated via spectral fitting in XSPEC. In the legend we report the name of each GRB.

**Supplementary Tables**

| GRB | z | $N_H(10^{22}\mathrm{cm}^{-2})$ | $T_i$(s) | $T_f$(s) |
|---|---|---|---|---|
| 090621A | - | 1.53 | 268 | 369 |
| 100619A | - | 0.46 | 88 | 168 |
| 110102A | - | 0.14 | 266 | 455 |
| 140512A | 0.725 | 0.12 | 124 | 216 |
| 161117A | 1.549 | 0.58 | 121 | 353 |
| 170906A | - | 0.23 | 90 | 194 |
| 180325A | 2.25 | 0.71 | 87 | 146 |
| 190604B | - | 0.21 | 219 | 262 |

**Supplementary Table 1: Main information about the GRBs of the first sample.** z is the redshift, when available. $N_H$ is the column density adopted in the spectral analysis of the X-ray tail. $T_i$ and $T_f$ are the central times of the initial and final bins of the spectral analysis, respectively.

| GRB | z | $N_H(10^{22}\mathrm{cm}^{-2})$ | $T_i$(s) | $T_f$(s) | $T_p^{\mathrm{BAT}}$(s) |
|---|---|---|---|---|---|
| 060729 | 0.54 | 0.03 | 133 | 163 | 93 |
| 060904A | - | 0.13 | 74 | 148 | 56 |
| 101023A | - | 0.17 | 91 | 180 | 63 |
| 120922A | - | 0.09 | 128 | 296 | 100 |
| 150323A | 0.593 | 0.44 | 153 | 241 | 136 |
| 160119A | - | 0.14 | 166 | 267 | 147 |
| 190106A | 1.86 | 0.68 | 99 | 227 | 76 |
| 190219A | - | 0.14 | 114 | 186 | 66 |

**Supplementary Table 2: Main information about the GRBs of the second sample.** z is the redshift, when available. $N_H$ is the column density adopted in the spectral analysis of the X-ray tail. $T_i$ and $T_f$ are the central times of the initial and final bins of the spectral analysis, respectively. $T_p^{\mathrm{BAT}}$ is the peak time of the BAT pulse preceding the X-ray tail, used for the extrapolation of $F_{\mathrm{max}}$.

| GRB | time (s) | $\alpha$ | $F_{(0.5-10)\ \mathrm{keV}}$ $(10^{-10}\mathrm{erg\ cm^{-2}s^{-1}})$ | cstat/dof |
|---|---|---|---|---|
| 090621A | | | | |
| | $265-271$ | $1.09^{+0.1}_{-0.1}$ | $397.6^{+19.7}_{-20.5}$ | $321.3/377$ |
| | $271-278$ | $1.05^{+0.11}_{-0.11}$ | $267.0^{+13.9}_{-14.6}$ | $282.7/351$ |
| | $278-289$ | $1.4^{+0.09}_{-0.09}$ | $138.8^{+6.0}_{-6.2}$ | $274.8/381$ |
| | $289-306$ | $1.6^{+0.06}_{-0.06}$ | $116.0^{+3.0}_{-3.1}$ | $509.3/548$ |
| | $306-339$ | $1.67^{+0.06}_{-0.06}$ | $52.6^{+1.5}_{-1.5}$ | $452.0/527$ |
| | $339-399$ | $2.03^{+0.1}_{-0.1}$ | $12.0^{+0.6}_{-0.6}$ | $253.7/346$ |
| 100619A | | | | |
| | $85-90$ | $0.69^{+0.09}_{-0.08}$ | $1311.0^{+77.8}_{-82.1}$ | $289.5/363$ |
| | $90-95$ | $1.06^{+0.08}_{-0.08}$ | $829.3^{+46.6}_{-49.1}$ | $259.4/347$ |
| | $95-101$ | $1.45^{+0.08}_{-0.08}$ | $454.1^{+23.8}_{-25.1}$ | $253.1/325$ |
| | $101-106$ | $1.64^{+0.09}_{-0.09}$ | $234.0^{+13.1}_{-13.7}$ | $233.5/277$ |
| | $106-120$ | $1.96^{+0.12}_{-0.12}$ | $130.0^{+8.4}_{-8.9}$ | $151.4/170$ |
| | $120-152$ | $2.38^{+0.08}_{-0.08}$ | $16.2^{+0.6}_{-0.6}$ | $267.6/320$ |
| | $152-185$ | $2.39^{+0.15}_{-0.16}$ | $4.7^{+0.4}_{-0.4}$ | $130.9/164$ |
| 110102A | | | | |
| | $260-272$ | $0.87^{+0.05}_{-0.05}$ | $484.3^{+20.5}_{-21.3}$ | $462.9/524$ |
| | $272-286$ | $1.19^{+0.05}_{-0.05}$ | $337.0^{+13.1}_{-13.6}$ | $463.2/485$ |
| | $286-314$ | $1.62^{+0.05}_{-0.05}$ | $107.0^{+3.8}_{-4.0}$ | $451.9/424$ |
| | $314-337$ | $2.12^{+0.05}_{-0.05}$ | $29.7^{+0.9}_{-0.9}$ | $324.3/386$ |
| | $337-387$ | $2.32^{+0.06}_{-0.06}$ | $9.1^{+0.3}_{-0.3}$ | $265.6/354$ |
| | $387-523$ | $2.39^{+0.08}_{-0.08}$ | $2.2^{+0.1}_{-0.1}$ | $272.9/299$ |

**Supplementary Table 3: Results of time resolved spectral analysis for the first sample of GRBs.** For each bin we report the time window, the photon index $\alpha$, the un-absorbed flux $F_{(0.5-10)\ \mathrm{keV}}$ and the statistics over the degrees of freedom (dof). The uncertainties are reported with $1\sigma$ level of confidence and they are calculated via spectral fitting in XSPEC.

| GRB | time (s) | $\alpha$ | $F_{(0.5-10)\ \mathrm{keV}}$ $(10^{-10}\mathrm{erg\ cm}^{-2}\mathrm{s}^{-1})$ | cstat/dof |
|---|---|---|---|---|
| 140512A | | | | |
| | $121-127$ | $0.39^{+0.08}_{-0.08}$ | $347.3^{+21.1}_{-22.1}$ | 279.3/414 |
| | $127-133$ | $0.73^{+0.08}_{-0.08}$ | $322.2^{+20.1}_{-21.2}$ | 271.6/359 |
| | $133-140$ | $1.16^{+0.06}_{-0.06}$ | $179.3^{+9.1}_{-9.5}$ | 388.3/416 |
| | $140-151$ | $1.06^{+0.05}_{-0.05}$ | $130.4^{+5.0}_{-5.1}$ | 482.6/530 |
| | $151-179$ | $1.58^{+0.04}_{-0.04}$ | $40.8^{+1.4}_{-1.4}$ | 459.8/480 |
| | $179-254$ | $2.19^{+0.05}_{-0.05}$ | $13.6^{+0.4}_{-0.4}$ | 383.2/419 |
| 161117A | | | | |
| | $118-123$ | $0.77^{+0.08}_{-0.08}$ | $1132.5^{+73.9}_{-77.8}$ | 295.9/358 |
| | $123-130$ | $0.85^{+0.07}_{-0.07}$ | $806.3^{+49.0}_{-51.4}$ | 320.9/371 |
| | $130-138$ | $1.02^{+0.06}_{-0.06}$ | $597.6^{+29.1}_{-30.5}$ | 320.6/440 |
| | $138-147$ | $1.16^{+0.06}_{-0.06}$ | $429.2^{+20.9}_{-21.6}$ | 343.5/427 |
| | $147-161$ | $1.44^{+0.05}_{-0.05}$ | $267.9^{+11.1}_{-11.5}$ | 378.2/422 |
| | $161-182$ | $1.71^{+0.04}_{-0.04}$ | $130.7^{+4.2}_{-4.4}$ | 399.9/458 |
| | $182-219$ | $1.91^{+0.09}_{-0.09}$ | $80.9^{+5.0}_{-5.4}$ | 190.8/202 |
| | $219-285$ | $2.17^{+0.04}_{-0.04}$ | $15.0^{+0.4}_{-0.4}$ | 390.1/465 |
| | $285-421$ | $2.39^{+0.06}_{-0.06}$ | $3.4^{+0.1}_{-0.1}$ | 290.3/335 |

**Supplementary Table 4: Results of time resolved spectral analysis for the first sample of GRBs-continued.** For each bin we report the time window, the photon index $\alpha$, the un-absorbed flux $F_{(0.5-10)\ \mathrm{keV}}$ and the statistics over the degrees of freedom (dof). The uncertainties are reported with $1\sigma$ level of confidence and they are calculated via spectral fitting in XSPEC.

| GRB | time (s) | $\alpha$ | $F_{(0.5-10)\ \mathrm{keV}}$ $(10^{-10}\mathrm{erg\ cm^{-2}s^{-1}})$ | cstat/dof |
|---|---|---|---|---|
| 170906A | | | | |
| | $88-93$ | $0.48^{+0.08}_{-0.08}$ | $1936.6^{+124.7}_{-132.6}$ | 264.0/331 |
| | $93-98$ | $0.82^{+0.08}_{-0.08}$ | $1277.8^{+78.1}_{-83.0}$ | 272.8/330 |
| | $98-105$ | $1.03^{+0.08}_{-0.08}$ | $729.9^{+45.3}_{-48.2}$ | 235.2/309 |
| | $105-110$ | $1.27^{+0.08}_{-0.08}$ | $601.6^{+35.4}_{-37.7}$ | 246.0/299 |
| | $110-116$ | $1.48^{+0.08}_{-0.08}$ | $325.3^{+18.4}_{-19.5}$ | 223.0/284 |
| | $116-123$ | $1.6^{+0.06}_{-0.07}$ | $329.1^{+15.0}_{-15.7}$ | 304.6/337 |
| | $123-129$ | $1.81^{+0.08}_{-0.08}$ | $240.6^{+12.6}_{-13.2}$ | 239.4/256 |
| | $129-139$ | $1.85^{+0.07}_{-0.07}$ | $178.0^{+8.4}_{-8.8}$ | 239.7/292 |
| | $139-152$ | $1.91^{+0.04}_{-0.04}$ | $76.9^{+2.1}_{-2.1}$ | 414.8/440 |
| | $152-174$ | $2.07^{+0.04}_{-0.04}$ | $45.5^{+1.2}_{-1.2}$ | 365.0/443 |
| | $174-215$ | $2.13^{+0.04}_{-0.04}$ | $22.7^{+0.6}_{-0.6}$ | 393.1/421 |
| 180325A | | | | |
| | $85-90$ | $0.76^{+0.07}_{-0.07}$ | $193.7^{+12.4}_{-13.2}$ | 262.1/384 |
| | $90-96$ | $0.87^{+0.07}_{-0.07}$ | $146.7^{+9.5}_{-10.1}$ | 289.9/349 |
| | $96-106$ | $1.16^{+0.07}_{-0.07}$ | $78.1^{+5.1}_{-5.3}$ | 237.4/325 |
| | $106-124$ | $1.84^{+0.08}_{-0.08}$ | $22.9^{+1.3}_{-1.4}$ | 267.5/274 |
| | $124-168$ | $2.03^{+0.08}_{-0.08}$ | $11.3^{+0.6}_{-0.7}$ | 229.5/268 |
| 190604B | | | | |
| | $216-222$ | $0.84^{+0.09}_{-0.09}$ | $1460.4^{+100.1}_{-107.2}$ | 247.5/297 |
| | $222-228$ | $1.01^{+0.09}_{-0.09}$ | $1144.3^{+84.0}_{-89.8}$ | 219.1/252 |
| | $228-234$ | $1.4^{+0.09}_{-0.09}$ | $619.1^{+40.2}_{-43.1}$ | 251.5/255 |
| | $234-243$ | $1.41^{+0.07}_{-0.07}$ | $477.2^{+26.5}_{-28.1}$ | 278.4/301 |
| | $243-254$ | $1.78^{+0.06}_{-0.06}$ | $201.5^{+8.0}_{-8.4}$ | 327.9/346 |
| | $254-270$ | $2.08^{+0.06}_{-0.06}$ | $87.8^{+3.0}_{-3.1}$ | 305.8/331 |

**Supplementary Table 5: Results of time resolved spectral analysis for the first sample of GRBs-continued.** For each bin we report the time window, the photon index $\alpha$, the un-absorbed flux $F_{(0.5-10)\ \mathrm{keV}}$ and the statistics over the degrees of freedom (dof). The uncertainties are reported with $1\sigma$ level of confidence and they are calculated via spectral fitting in XSPEC.

| GRB | time (s) | $\alpha$ | $F_{(0.5-10)\ \mathrm{keV}}$ $(10^{-10}\mathrm{erg\ cm^{-2}s^{-1}})$ | cstat/dof |
|---|---|---|---|---|
| 060729 | | | | |
| | $130-135$ | $1.95^{+0.06}_{-0.07}$ | $481.9^{+23.0}_{-24.4}$ | 266.9/265 |
| | $135-141$ | $2.23^{+0.08}_{-0.07}$ | $323.7^{+15.8}_{-16.0}$ | 252.8/221 |
| | $141-147$ | $2.37^{+0.08}_{-0.08}$ | $213.9^{+10.2}_{-10.6}$ | 220.7/202 |
| | $147-152$ | $2.63^{+0.07}_{-0.08}$ | $131.9^{+5.5}_{-5.7}$ | 234.4/197 |
| | $152-159$ | $2.74^{+0.08}_{-0.08}$ | $77.0^{+3.1}_{-3.2}$ | 232.0/203 |
| | $159-167$ | $2.82^{+0.07}_{-0.07}$ | $57.1^{+1.9}_{-1.9}$ | 193.1/246 |
| 060904A | | | | |
| | $72-77$ | $1.09^{+0.08}_{-0.08}$ | $257.2^{+17.2}_{-18.2}$ | 245.7/294 |
| | $77-82$ | $1.19^{+0.08}_{-0.09}$ | $220.5^{+15.8}_{-16.7}$ | 233.5/256 |
| | $82-88$ | $1.45^{+0.09}_{-0.09}$ | $112.9^{+8.1}_{-8.7}$ | 172.7/239 |
| | $88-97$ | $1.55^{+0.05}_{-0.05}$ | $71.0^{+3.0}_{-3.1}$ | 387.7/401 |
| | $97-109$ | $1.69^{+0.05}_{-0.05}$ | $51.2^{+2.0}_{-2.1}$ | 369.1/378 |
| | $109-128$ | $1.98^{+0.06}_{-0.06}$ | $27.6^{+1.1}_{-1.1}$ | 320.2/345 |
| | $128-168$ | $2.37^{+0.06}_{-0.06}$ | $11.1^{+0.4}_{-0.4}$ | 249.3/298 |
| 101023A | | | | |
| | $88-94$ | $1.29^{+0.08}_{-0.08}$ | $294.0^{+18.7}_{-19.9}$ | 252.9/326 |
| | $94-99$ | $1.35^{+0.07}_{-0.07}$ | $227.6^{+13.1}_{-13.8}$ | 289.7/331 |
| | $99-105$ | $1.41^{+0.08}_{-0.08}$ | $178.4^{+11.0}_{-11.6}$ | 244.6/304 |
| | $105-110$ | $1.47^{+0.07}_{-0.07}$ | $165.7^{+9.2}_{-9.7}$ | 298.3/319 |
| | $110-118$ | $1.58^{+0.08}_{-0.08}$ | $109.9^{+6.4}_{-6.8}$ | 255.5/309 |
| | $118-127$ | $1.81^{+0.08}_{-0.08}$ | $85.0^{+4.7}_{-5.0}$ | 280.8/291 |
| | $127-143$ | $1.92^{+0.05}_{-0.05}$ | $50.8^{+1.6}_{-1.6}$ | 417.0/429 |
| | $143-168$ | $2.21^{+0.05}_{-0.05}$ | $28.6^{+0.8}_{-0.8}$ | 341.2/399 |
| | $168-193$ | $2.23^{+0.07}_{-0.07}$ | $15.3^{+0.6}_{-0.6}$ | 271.6/311 |

**Supplementary Table 6: Results of time resolved spectral analysis for the second sample of GRBs.** For each bin we report the time window, the photon index $\alpha$, the un-absorbed flux $F_{(0.5-10)\ \mathrm{keV}}$ and the statistics over the degrees of freedom (dof). The uncertainties are reported with $1\sigma$ level of confidence and they are calculated via spectral fitting in XSPEC.

| GRB | time (s) | $\alpha$ | $F_{(0.5-10)\ \mathrm{keV}}$ $(10^{-10}\mathrm{erg\ cm^{-2}s^{-1}})$ | cstat/dof |
|---|---|---|---|---|
| 120922A | | | | |
| | $125-130$ | $1.09^{+0.07}_{-0.07}$ | $374.2^{+22.8}_{-24.2}$ | $297.7/333$ |
| | $130-136$ | $1.19^{+0.07}_{-0.07}$ | $348.1^{+21.4}_{-22.6}$ | $304.4/321$ |
| | $136-141$ | $1.32^{+0.08}_{-0.08}$ | $251.9^{+16.9}_{-18.0}$ | $268.3/281$ |
| | $141-146$ | $1.28^{+0.07}_{-0.07}$ | $292.0^{+17.4}_{-18.3}$ | $280.1/316$ |
| | $146-152$ | $1.46^{+0.08}_{-0.08}$ | $203.0^{+13.3}_{-14.1}$ | $234.1/276$ |
| | $152-157$ | $1.53^{+0.07}_{-0.07}$ | $186.4^{+10.5}_{-11.0}$ | $263.6/294$ |
| | $157-162$ | $1.58^{+0.07}_{-0.07}$ | $168.2^{+9.4}_{-9.8}$ | $274.1/299$ |
| | $162-170$ | $1.64^{+0.07}_{-0.07}$ | $131.0^{+7.6}_{-8.0}$ | $239.0/286$ |
| | $170-178$ | $1.77^{+0.06}_{-0.06}$ | $97.7^{+4.8}_{-5.1}$ | $291.7/294$ |
| | $178-188$ | $1.82^{+0.07}_{-0.07}$ | $80.2^{+4.0}_{-4.2}$ | $250.9/297$ |
| | $188-199$ | $1.91^{+0.06}_{-0.06}$ | $65.7^{+3.0}_{-3.2}$ | $288.6/300$ |
| | $199-212$ | $1.9^{+0.07}_{-0.07}$ | $62.1^{+3.2}_{-3.3}$ | $233.5/282$ |
| | $212-228$ | $1.84^{+0.04}_{-0.04}$ | $50.9^{+1.5}_{-1.6}$ | $390.6/436$ |
| | $228-247$ | $1.9^{+0.04}_{-0.04}$ | $43.6^{+1.2}_{-1.3}$ | $341.0/444$ |
| | $247-276$ | $1.99^{+0.04}_{-0.04}$ | $28.0^{+0.8}_{-0.8}$ | $375.0/428$ |
| | $276-316$ | $2.2^{+0.04}_{-0.04}$ | $19.1^{+0.5}_{-0.5}$ | $320.7/399$ |
| 150323A | | | | |
| | $150-156$ | $1.73^{+0.07}_{-0.07}$ | $333.5^{+17.6}_{-18.6}$ | $238.1/282$ |
| | $156-161$ | $1.8^{+0.09}_{-0.09}$ | $211.7^{+12.4}_{-13.1}$ | $202.8/254$ |
| | $161-167$ | $1.92^{+0.08}_{-0.08}$ | $149.6^{+7.8}_{-8.3}$ | $251.2/276$ |
| | $167-176$ | $2.19^{+0.08}_{-0.08}$ | $86.4^{+3.9}_{-4.0}$ | $254.5/264$ |
| | $176-190$ | $2.23^{+0.07}_{-0.07}$ | $66.9^{+2.7}_{-2.8}$ | $242.1/300$ |
| | $190-215$ | $2.53^{+0.05}_{-0.05}$ | $29.0^{+0.7}_{-0.7}$ | $300.3/364$ |
| | $215-267$ | $2.98^{+0.06}_{-0.07}$ | $10.2^{+0.3}_{-0.3}$ | $251.2/298$ |

**Supplementary Table 7: Results of time resolved spectral analysis for the second sample of GRBs-continued.** For each bin we report the time window, the photon index $\alpha$, the un-absorbed flux $F_{(0.5-10)\ \mathrm{keV}}$ and the statistics over the degrees of freedom (dof). The uncertainties are reported with $1\sigma$ level of confidence and they are calculated via spectral fitting in XSPEC.

| GRB | time (s) | $\alpha$ | $F_{(0.5-10)\ \mathrm{keV}}$ $(10^{-10}\mathrm{erg\ cm^{-2}s^{-1}})$ | cstat/dof |
|---|---|---|---|---|
| 160119A | | | | |
| | $164-169$ | $1.25^{+0.07}_{-0.07}$ | $183.1^{+10.9}_{-11.4}$ | $279.0/347$ |
| | $169-174$ | $1.37^{+0.08}_{-0.08}$ | $171.7^{+10.7}_{-11.4}$ | $241.7/303$ |
| | $174-180$ | $1.47^{+0.07}_{-0.07}$ | $134.6^{+7.9}_{-8.4}$ | $219.9/320$ |
| | $180-185$ | $1.54^{+0.07}_{-0.07}$ | $109.8^{+6.3}_{-6.6}$ | $217.4/309$ |
| | $185-193$ | $1.66^{+0.05}_{-0.05}$ | $129.6^{+5.3}_{-5.5}$ | $323.5/389$ |
| | $193-200$ | $1.63^{+0.06}_{-0.06}$ | $126.9^{+5.6}_{-5.9}$ | $341.0/376$ |
| | $200-209$ | $1.8^{+0.06}_{-0.06}$ | $93.4^{+3.9}_{-4.1}$ | $347.9/370$ |
| | $209-223$ | $1.94^{+0.06}_{-0.06}$ | $63.6^{+2.5}_{-2.6}$ | $324.0/342$ |
| | $223-242$ | $2.12^{+0.06}_{-0.06}$ | $37.9^{+1.5}_{-1.5}$ | $292.8/326$ |
| | $242-291$ | $2.43^{+0.07}_{-0.07}$ | $13.8^{+0.5}_{-0.5}$ | $249.1/308$ |
| 190106A | | | | |
| | $96-101$ | $1.56^{+0.08}_{-0.08}$ | $221.9^{+13.4}_{-14.4}$ | $247.0/263$ |
| | $101-106$ | $2.02^{+0.09}_{-0.09}$ | $120.6^{+7.5}_{-7.9}$ | $226.2/220$ |
| | $106-114$ | $2.31^{+0.1}_{-0.1}$ | $67.7^{+3.8}_{-4.0}$ | $150.5/198$ |
| | $114-130$ | $2.55^{+0.06}_{-0.06}$ | $33.2^{+1.0}_{-1.0}$ | $271.4/319$ |
| | $130-181$ | $2.69^{+0.07}_{-0.07}$ | $9.6^{+0.3}_{-0.3}$ | $359.4/332$ |
| | $181-274$ | $2.38^{+0.09}_{-0.09}$ | $2.6^{+0.1}_{-0.1}$ | $242.7/268$ |
| 190219A | | | | |
| | $111-117$ | $1.99^{+0.08}_{-0.08}$ | $99.8^{+5.3}_{-5.6}$ | $199.8/251$ |
| | $117-124$ | $2.29^{+0.07}_{-0.07}$ | $116.9^{+5.0}_{-5.1}$ | $218.2/267$ |
| | $124-134$ | $2.46^{+0.07}_{-0.07}$ | $82.8^{+3.4}_{-3.5}$ | $238.2/254$ |
| | $134-146$ | $2.51^{+0.09}_{-0.09}$ | $46.0^{+2.3}_{-2.3}$ | $188.4/215$ |
| | $146-164$ | $2.67^{+0.05}_{-0.05}$ | $33.8^{+0.9}_{-0.9}$ | $328.2/316$ |
| | $164-208$ | $3.05^{+0.06}_{-0.06}$ | $13.8^{+0.4}_{-0.4}$ | $295.2/306$ |

**Supplementary Table 8: Results of time resolved spectral analysis for the second sample of GRBs-continued.** For each bin we report the time window, the photon index $\alpha$, the un-absorbed flux $F_{(0.5-10)\ \mathrm{keV}}$ and the statistics over the degrees of freedom (dof). The uncertainties are reported with $1\sigma$ level of confidence and they are calculated via spectral fitting in XSPEC.

## References


1. Fenimore, E. E., Madras, C. D., Nayakshin, S. Expanding relativistic shells and gamma-ray burst temporal structure. *Astrophys. J.* **473**, 998 (1996)

2. Dermer, C. D. Curvature effects in gamma-ray burst colliding shells. *Astrophys. J.* **614**, 284 (2004)

3. Genet, F., Granot, J. Realistic analytic model for the prompt and high-latitude emission in GRBs. *Mon. Not. R. Astron. Soc.* **399**, 1328 (2009)

4. Salafia, O. S., Ghisellini, G., Pescalli, A., Ghirlanda, G., Nappo, F. Light curves and spectra from off-axis gamma-ray bursts. *Mon. Not. R. Astron. Soc.* **461**, 3607 (2016)

5. Uhm, Z. L., Zhang, B. On the curvature effect of a relativistic spherical shell. *Astrophys. J.* **808**, 33 (2015)

6. Uhm, Z. L., Zhang, B. Evidence of bulk acceleration of the GRB X-ray flare emission region. *Astrophys. J.* **824**, L16 (2016)

7. Uhm, Z. L., Zhang, B. Toward an understanding of GRB prompt emission mechanism. I. The origin of spectral lags. *Astrophys. J.* **825**, 97 (2016)

8. Uhm, Z. L., Zhang, B., Racusin, J. Toward an understanding of GRB prompt emission mechanism. II. Patterns of peak energy evolution and their connection to spectral lags. *Astrophys. J.* **869**, 100 (2018)

9. Lyutikov, M., Blandford, R. Gamma bay bursts as electromagnetic outflows. arXiv e-prints astro-ph/0312347 (2003)

10. Narayan, R., & Kumar, P. A turbulent model of gamma-ray burst variability. *Mon. Not. R. Astron. Soc.* **394**, L117 (2009)

11. Barniol Duran, R., Leng, M., Giannios, D. An anisotropic minijets model for the GRB prompt emission. *Mon. Not. R. Astron. Soc.* **455**, L6 (2016)

12. Geng, J.-J., Huang, Y.-F., & Dai, Z.-G. Steep eecay of GRB X-ray flares: the results of anisotropic synchrotron radiation. *Astrophys. J. Lett.* **841**, L15 (2017)

13. Rees, M. J., Meszaros, P. Unsteady outflow models for cosmological gamma-ray bursts. *Astrophys. J.* **430**, L93 (1994)

14. Drenkhahn, G., Spruit, H. C. Efficient acceleration and radiation in Poynting flux powered GRB outflows. *Astron. Astrophys.* **391**, 1141 (2002)

15. Pe'er, A. Temporal evolution of thermal emission from relativistically expanding plasma. *Astrophys. J.* **682**, 463 (2008)

16. Piran, T. Gamma-ray bursts and the fireball model. *Phys. Rep.* **314**, 575 (1999)

17. Kumar, P., Narayan, R., & Johnson, J. L. Properties of gamma-ray burst progenitor stars. *Science* **321**, 376 (2008)

18. Hascoët, R., Daigne, F., Mochkovitch, R. Accounting for the XRT early steep decay in models of the prompt gamma-ray burst emission. *Astron. Astrophys.* **542**, L29 (2012)

19. Beloborodov, A. M. Radiative transfer in ultrarelativistic outflows. *Astrophys. J.* **737**, 68 (2011)

20. Asano, K., Terasawa, T. Slow heating model of gamma-ray burst: photon spectrum and delayed emission. *Astrophys. J.* **705**, 1714 (2009)

21. Asano, K. & Terasawa, T. Stochastic acceleration model of gamma-ray burst with decaying turbulence. *Mon. Not. R. Astron. Soc.* **454**, 2242 (2015)

22. Panaitescu, A. Adiabatic and radiative cooling of relativistic electrons applied to synchrotron spectra and light curves of gamma-ray burst pulses. *Astrophys. J.* **886**, 106 (2019)
